# High number of diarrhoeal co-infections in travellers to Benin, West Africa

**DOI:** 10.1186/1471-2334-14-81

**Published:** 2014-02-12

**Authors:** Tinja Lääveri, Sari H Pakkanen, Jenni Antikainen, Jukka Riutta, Sointu Mero, Juha Kirveskari, Anu Kantele

**Affiliations:** 1 Division of Infectious Diseases, Department of Medicine, Helsinki University Central Hospital, PO Box 348, 00029 HUS Helsinki, Finland; 2 Department of Bacteriology and Immunology, Haartman Institute, University of Helsinki, PO Box 2100014 Helsinki, Finland; 3 Helsinki University Hospital Laboratory (HUSLAB), Bacteriology, PO Box 400, 00029 HUS Helsinki, Finland; 4 Aava Travel Clinic, Medical Centre Aava, Annankatu 34, 00100 Helsinki, Finland; 5 Department of Clinical Medicine, University of Helsinki, PO Box 20, 00014 Helsinki, Finland

**Keywords:** Traveller’s diarrhoea, TD, EAEC, ETEC, EPEC, West Africa, qPCR, Campylobacter, Salmonella

## Abstract

**Background:**

Travellers’ diarrhoea (TD) is the most frequent health problem among travellers to the tropics. Using routine techniques, the aetiology mostly remains unresolved, whereas modern molecular methods enable reducing the number of equivocal cases considerably. While many studies address the aetiology of TD in Asian, Central American and North African tourist resorts, only few focus on Western Africa.

**Methods:**

Stool samples from 45 travellers travelling in Benin, West Africa, were analyzed by a new multiplex qPCR assay for Salmonella, Yersinia, Campylobacter, *Vibrio cholerae,* Shigella or enteroinvasive (EIEC), enterohaemorrhagic (EHEC), enterotoxigenic (ETEC), enteroaggregative (EAEC), and enteropathogenic *Escherichia coli* (EPEC).

**Results:**

All 18 pre-travel samples proved negative for bacterial pathogens. Of the 39/45 (87%) travellers having had TD, EPEC was detected in post-travel samples in 30 (77%) cases, EAEC in 23 (59%), ETEC in 22 (56%), Shigella or EIEC in 7 (18%), EHEC in two (5%), and Salmonella in one (3%). In 31(79%) of the TD cases two or more bacterial pathogens were identified. Two (8%) samples remained negative: both patients had taken antimicrobials for TD.

**Conclusions:**

EPEC, EAEC and ETEC were the most common findings. 79% of the cases had a co-infection. As modern diagnostics reveals in most patients a multitude of pathogens, the role of each pathogen should be re-evaluated.

## Background

Travellers’ diarrhoea (TD) has been estimated to be contracted by 80 million individuals every year [1], yet the causative agents often remain poorly characterized. Although TD appears to be of bacterial origin in as many as 75% of the cases [2,3], the causative microorganism is left unidentified in up to half of those reported [2-5]. When using modern PCR-based methods, diarrhoeagenic *E. coli* has been detected in one third of the culture-negative stool samples [6]. Recently, researchers employing more advanced diagnostic techniques have succeeded in decreasing the number of unexplained TD cases to 5-24% [1,7,8].

Enterotoxigenic *Escherichia coli* (ETEC) has been considered the most common causative agent for TD worldwide [2,3]. With improved methodology, however, enteroaggregative *E. coli* (EAEC) has been reported even more often than ETEC [1,8,9]. According to some recent studies [1,7,8], multiple pathogens are detected more frequently (up to 60% of cases) than anticipated before.

In many laboratories the methods available for routine clinical analyses will only identify Salmonella, Campylobacter, Shigella and Yersinia, and *Clostridium difficile* or certain variants of EHEC are investigated on separate request. This approach leaves all patients having the most common TD pathogen, diarrhoeagenic *E. coli*, without accurate bacteriological explanation for their disease. We have recently described a new multiplex qPCR method which allows rapid detection of nine diarrhoeal pathogens at the same time [1]. This method was adopted into routine use at our laboratory in 2012.

While earlier microbial research centred on travellers to Southeast Asia, South Asia, Central America, and Northern Africa [3], other parts of Africa have been given scarce attention. We know of merely five studies [8,10-13], just two [8,13] of them from this century, describing the aetiology of TD in travellers to Central or Western Africa. In these reports the percentage of unexplained cases ranges between 5 and 60.

The present study looks into the aetiology of TD using modern methods and focusing on a group travelling together under similar conditions in a non-touristic destination in Sub-Saharan Africa.

## Methods

### Study population/recruitment

**Figure 1:**
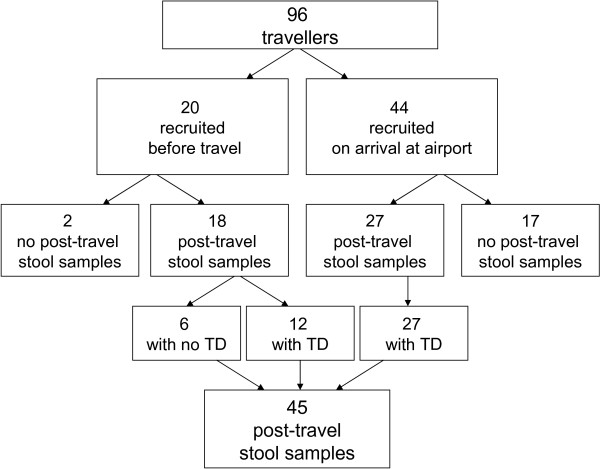
The study protocol.

The participants were recruited from among 96 travellers (Figure 1) destined for Grand Popo, Benin, West Africa in November 2009. They were each given questionnaires and test tubes for stool samples either at an information session or a health care appointment before the journey; they also received brochures on pertinent health issues, including detailed instructions on preventive measures against TD. The participants were asked to provide stool samples prior to departure and on return, and to fill in the questionnaires before (Q1) and immediately after (Q2) the trip, and a third one (Q3) three weeks later. As the initial number of volunteers providing Q1 and pre-travel stool samples proved quite low (20/96), the researchers went to meet the returning travellers at the airport not only to remind the volunteers of the study but also to ask for new ones to participate. On that occasion 44 new subjects were included in the research despite lacking pre-travel samples, 17 of whom failed to return both stool sample and questionnaire. Of the initial 20 participants who had provided the first faecal sample before the journey, two (10%) did not give the second one after it. The 19 subjects (2 recruited at start, 17 at airport) failing to provide post-travel samples were considered dropouts. Of the 45 who gave a second stool sample and answered Q2, 27 also returned Q3. Thus, 18 pre-travel and 45 post-travel samples were analyzed for pathogens with qPCR.

### The journey in brief

The group travelled together for 9 days before returning to Finland. They were accommodated at a residence and three nearby hotels. Meals were eaten in smaller groups at local restaurants.

### Definition and classification of travellers’ diarrhoea

Travellers’ diarrhoea was defined according to the WHO criteria [14] as the passage of 3 or more loose or liquid stools per day, or more frequently than is normal for the individual. Severe TD was defined as six or more unformed diarrhoeal stools per 24 hours or diarrhoea accompanied by fever or containing blood, or requiring hospitalization, and mild TD as 1–2 unformed daily stools. Cases not fulfilling these criteria were classified as moderate. Diarrhoea was defined as travel-related had it begun abroad or within seven days after returning home.

### The questionnaires

The subjects filled in three questionnaires: pre-travel questionnaire (Q1) covered demographic particulars, current medical conditions, knowledge about travel-related risks, vaccination status and possible diarrhoeal symptoms at the time of first (pre-travel) faecal sample. Questionnaire 2 (Q2) was collected on return to Finland. The questions in Q2 concerned diarrhoeal and other symptoms during and just after the trip, medications taken over its course, risk behaviour, and contacts with local health care. The third questionnaire (Q3) collected three weeks after return was a follow-up focusing on symptoms over the first three weeks at home, possible medications, and contacts with health care.

Adherence to TD prevention advice was measured in Q2 by the following questions: “Did you drink tap water, purified water or bottled water?”, “Did you eat cold salads”, “Did you eat uncooked meat or fish?”, “Did you always/often/seldom wash hands before eating?” and “Did you use utensils/bare fingers?”

### Collection of stool samples

The volunteers were asked to give a pre-travel stool sample within one week before departure and a post-travel sample from the first (or second) stool passed after returning home. The samples were collected as swabs in Copan M40 Transystem tubes (Copan Diagnostics, Brescia, Italy) and sealed in special mailing envelopes. As the group returned to Finland on a Saturday evening, they were advised to refrigerate their post-travel samples and mail them only on Monday morning; the samples thus reached the laboratory in 1–3 days. There the swabs were frozen in -20°C for later analysis.

### Nucleid acid extraction

The extraction process and qPCR have been recently described in detail [1]. Briefly, total nucleid acids were extracted using the standard semi-automated protocol of easyMAG (bioMérieux, Marcy-l’Etoile, France). The swap was thawed and directly inoculated into 2 mL of lysis buffer, and processed according to manufacturer’s instructions. A general extraction protocol with 25 μL elution volume was performed. A 0.5 μL of eluted template was utilized per each qPCR reaction in 20 μL final volume.

### qPCR analysis of bacterial pathogens

The analyses were carried out with a multiplex qPCR method [1] which covers the following pathogens: diarrhoeagenic *E. coli* including EPEC, ETEC, EAEC, EHEC and EIEC or Shigella as well as Salmonella, Yersinia, *Vibrio cholerae,* and *Campylobacter spp.* This assay described in detail recently [1] allows a rapid and simultaneous examination of all these pathogens, providing results in just four hours.

We also investigated the bundle-forming pilus structural gene (bfpA) linked to the virulence of typical EPEC. The gene was amplified using the primers F_bfpA_001 CTGTCTTTGATTGAATCTGCAATGG and R_bfpA_001CTGAAATAGCATTCTGTGACTTATTGG. The detection was performed with the Stratagene MxPro 3005P instrument (Agilent Technologies, Inc., CA) utilizing SYBR Green chemistry (Thermo Fisher Scientific, Finland) by a standard two-step protocol with melting curve analysis. Briefly, initial denaturation time of 15 min was followed by 45 cycles of denaturation at 94°C for 1 minute, and annealing/extension at 60°C for 1 min. The PCR amplicon was confirmed correct by Sanger sequencing and characteristic melting temperature.

### Statistical analysis

Differences between the various traveller groups were examined by Chi-square tests using SPSS 19.0.0.1 (SPSS Inc., Chicago, IL, USA).

### Ethical clearance and informed patient consent

This study was conducted in accordance with the ethical principles of the Declaration of Helsinki and the protocol approved by the Ethics Committee of the Department of Medicine in Helsinki University Central Hospital. Written informed consent was obtained before enrolment from all patients and volunteers, and legal caretakers of minors.

## Results

### Demographics of the study population

Background information on the volunteers is provided in Table 1. Of the 45 study subjects, 27 (60%) were female. The average age was 46 years (range 15 – 68 years). Seven had medication for an underlying chronic illness (hypertension, diabetes, hypercholesterolaemia).

**Table 1:** Background data of 45 travellers to Benin, West Africa

	**Yes n (%)**	**No n (%)**	**Data not available n (% of all)**
Sex: female (%)	27 (60)	18 (40)	0
Self-reported good knowledge about travel-related risks (Q1)	34 (77)	0	11 (23)
Underlying chronic disease (Q1)	7 (16)	38 (84)	0
Appropriate antimalarial prophylaxis* (Q2)	42 (93)	2 (4)	1 (2)
Pre-travel vaccination status (Q1)			
Yellow fever*	40 (89)	0	5 (11)
Tetanus + diphtheria*	36 (80)	0	9 (20)
Polio*	21 (64)	12 (36)	12 (27)
MMR*,**	19 (42)	1 (2)	15 (33)
Hepatitis A*	39 (87)	0	6 (13)
Typhoid fever*** (either peroral or injectable)	4 (9)	10 (22)	31 (69)
Meningococcal*** (type not specified)	2 (4)	9 (20)	34 (76)
Cholera***	3 (7)	10 (22)	32 (71)

*National guidelines recommend to all travellers to Benin [16].

**Either has a history of mumps, measles and rubella or has received two doses of MMR vaccine.

***Vaccinations according to risk assessment [16].

### GI and other symptoms in the study population

Of the 45 subjects, 39 (87%) had contracted TD over the journey (Figure 1). 14 (36%) cases proved severe, 19 (49%) moderate, and 6 (15%) mild. Severe TD was accompanied by fever in 11/39 (28%) cases. Only two reported having contacted local healthcare, and none was hospitalized. TD started on average on the 6th day of travel, and in 50% of the cases the symptoms lasted for over three days. Of the 18 initially recruited who had returned both pre- and post-travel samples, 12 (67%) had developed TD and six (33%) had remained asymptomatic, whereas all the 27 recruited at the airport had had TD over the journey. 37/39 volunteers (95%) had ongoing symptoms at the time of sampling. The third questionnaire (Q3) was returned by 27 study subjects: two with TD (7%) still had slightly loose stools, 22 (81%) no longer had any symptoms, and three (11%) initially asymptomatic had not developed symptoms over the follow-up either.

### Microbiological findings

All 18 pre-travel samples were tested negative for the nine bacterial pathogens. The results of post-travel sample analyses are provided in Table 2. Only 3/45 post-travel stools proved negative. 35/45 (78%) subjects were identified to have contracted two or more pathogens; 31/39 (79%) of those with TD had multiple pathogens. EPEC (76%) was the most common finding followed by EAEC (60%) and ETEC (56%). No differences were found between symptomatic and asymptomatic subjects in the relative proportions of the various pathogens. 11/34 (32%) of EPEC strains proved bfpA-positive.

**Table 2:** Bacteriological findings related to TD symptoms over the course of the journey in 45 travellers

	**All n = 45 (% of all)**	**Asymptomatic n = 6 (% of these)**	**TD n = 39 (% of these)**	**Fever n = 11 (% of these)**
EPEC	34 (76)	4 (67)	30 (77)	6 (55)
EAEC	27 (60)	4 (67)	23 (59)	7 (64)
ETEC	25 (56)	3 (50)	22 (56)	6 (55)
EIEC/Shigella	7 (16)	0	7 (18)	5 (45)*
EHEC	2 (4)	0	2 (5)	0 (0)
Salmonella	1 (2)	0	1 (3)	0 (0)
Campylobacter	0	0	0	0
*Vibrio cholerae*	0	0	0	0
Yersinia spp	0	0	0	0
No pathogen	3 (7)	0	3 (8)	1 (9)
Single pathogen	7 (16)	2 (33)	5 (13)	2(18)
Multiple pathogens	35 (78)	4 (67)	31(79)	8 (73)
3 or more pathogens	15 (33)	1 (17)	14 (36)	4 (36)

All subjects with fever also had TD. The figures are given separately for each traveller, and for the groups with or without TD. Differences between the two groups were analyzed with Chi-square tests and indicated with asterisks when proved significant.

All differences were statistically insignificant except *p = 0,006.

### Antimicrobial medications

Data on the use of antimicrobials are provided in Table 3. Consistent with the Finnish recommendations [15], none of the travellers had used antimicrobials for prophylaxis of TD.; 22 had taken doxycycline as antimalarial chemoprophylaxis. Six (15%) subjects had taken antimicrobials against TD during the trip: 4 had used ciprofloxacin for 1–3 days, one azithromycin for 2 days and one penicillin (sic!), but did not report the number of days. After returning home, 8 more took a course of antimicrobial medication. Two were prescribed ciprofloxacin at the travel clinic 3–10 days after the journey, as their symptoms had not resolved; four with ongoing symptoms had EIEC/Shigella in their stool samples, and were treated with ciprofloxacin, adhering to the Finnish recommendation that all Shigella cases need to be treated because of its low infectious dose; one took ciprofloxacin for a urinary tract infection; one already asymptomatic self-administered ciprofloxacin despite being told that no medication was needed for the EAEC detected in her post-travel stool sample.

**Table 3:** Medication used by travellers during the journey

	**TD group n =39 (% of these)**	**Asymptomatic group n = 6 (% of these)**
Loperamide	19 (49)	0 (0)
Probiotics* (p = 0,632)	18 (69)	4 (66)
Proton Pump Inhibitors (p = 0,292)	2 (5)	1 (17)
Anticrobial medication during travel (excluding doxycycline)	6** (15)	0 (0)
Anticrobial medication after travel (excluding doxycycline)	8*** (21)	0 (0)
Doxycycline as antimalarial (p = 0,349)	18 (46)	4 (67)

The figures are given separately for those with or without TD. Differences between these two groups were statistically insignificant when analyzed with chi-square tests.

*Information missing from 13 travellers; % calculated on the basis of data from others.

**4 took ciprofloxacin, 1 azithromycin and 1 penicillin (sic!).

***all took ciprofloxacin, one for UTI, 7 for TD.

### Risk behaviour among the subjects

The travellers considered themselves aware of all the health-related risks e.g. malaria and TD, and most of them had been vaccinated according to national recommendations [16]. Despite this, not more than 31% of the subjects with TD and 33% of the asymptomatic actually followed all TD prevention advice (see Table 4).

**Table 4:** Adherence to TD prevention advice

	**TD group n = positive/information available (% of these)**	**Asymptomatic group n = positive/information available (% of these)**
Ignored any “TD prevention advice”	28/38 (69)	4/6 (67)
Drank only bottled water	38/39 (97)	6/6 (100)
Ate cold salads	24/38 (63)	4/6 (67)
Ate uncooked meat or fish	3/15 (20)	1/2 (50)
Consumed alcohol 3 or more units/day	19/33 (58)	4/6 (67)
Ate without utensils (bare fingers)	10/39 (26)	2/6 (33)
Washed hands only seldom	3/39 (8)	1/6 (17)

The figures are given separately for those with and without TD. Differences between the two groups were statistically insignificant when analyzed with Chi-square tests.

## Discussion

In addition to providing a wide-range analysis of bacterial pathogens in TD, the present study contributes to the limited knowledge of diarrhoeal pathogens in travellers to West Africa.

There are no previous reports of TD in tourists to Benin, and very few of travellers to West Africa [8,10-13] One of the noteworthy findings was the exceptionally high incidence of TD among our study subjects. Although Sub-Saharan Africa is generally presented as an area with a 20 – 60% risk for TD [17-20], 87% of our travellers reported having contracted the disease. However, part of our group were recruited at the airport only after the journey. All these volunteers had TD and, in fact, their symptoms appeared somewhat more severe than those of the prospectively recruited TD patients (data not shown); the symptoms presumably encouraged them to take part in the study. The actual proportion of patients with TD should, therefore, be evaluated from those who agreed to participate at baseline: 60% (12/20) of them had TD. Interestingly, the proportion of cases with ETEC appeared higher among those recruited at the airport (67%) than those enrolled prospectively (33%), correlating with the exceptionally virulent nature of ETEC and severity of symptoms.

In addition to the incidence of TD being high among our travellers, one third of the cases proved severe. Their proportion appears higher than reported elsewhere, yet the criteria for severe disease vary between studies [18,19]. The severity of the clinical picture presumably explains the comparatively high (15%) use of antimicrobials for TD over the journey and also afterwards (18% for TD). In two recent investigations conducted among Dutch [18] and Swiss [19] travellers, only 5 and 7% used antimicrobials for TD, yet, in these studies only 4 and 8% of the TD cases were considered severe.

Even though the travellers considered themselves well-informed of TD as a health risk, barely one third of them followed the TD prevention advice given. The high number of TD cases among these travellers could be ascribed to this, yet poor adherence is very common (60 – 95%) among tourists in general [18,20-22]. Moreover, even strictly followed preventive measures appear not to succeed in preventing TD [18-22].

While traditional routine diagnostics in most countries only covers a few bacterial pathogens, modern PCR methods have become increasingly applicable alongside [1]. Indeed, our assay has been successfully used in routine diagnostics of TD in the Helsinki capital area since 2012. As obvious, even these approaches cannot be designed to cover more than a limited number of pathogens. Some authors [7] have suggested that the unresolved TD cases could be ascribed to microbes earlier not recognized as causing TD. In our study using modern diagnostic methods, the proportion of unexplained TD was as low as 8%. Furthermore, the two TD patients with negative stool samples had taken a course of ciprofloxacin and had their symptoms go away by the time of the stool sampling. We thus identified a pathogen in all patients with symptoms at the time of sampling. The data are consistent with earlier studies where PCR-based methods have substantially decreased the number of unexplained cases [1,6,8].

The selection of TD pathogens contracted in Benin was of special interest. A large variety of pathogens was detected with the various diarrhoeagenic *E. coli*s as the most prevalent findings. Among the nine bacteria studied, EPEC, EAEC and ETEC proved most frequent, followed by EIEC/Shigella and Salmonella. No cases with *Vibrio cholerae*, Yersinia or Campylobacter were found. Campylobacter, although common in Southeast Asia and Nepal [3,5,23,24], has not been considered a major pathogen in West Africa in previous reports either [8,10-13,24]. *Vibrio cholerae* and Yersinia are rare in TD everywhere. Little is known about the aetiology of childhood diarrhoea in Benin; no data from there was included even in a recent report centring on Africa and Asia [25]. Molecular biology methods are not currently feasible for routine diagnostics in developing countries, yet periodic microbial surveillance for diarrhoea is considered a necessity today [25,26].

It is noteworthy that, while EPEC is one of the most frequent findings in childhood diarrhoea in developing countries [27], its significance as a TD pathogen among adults has not been confirmed [5,28]. In our patients, of whom all but one were adults, 32% of the EPEC strains proved bfpA-positive by PCR, and were thus considered to represent typical EPEC serotypes [25,27]. No difference was seen in the occurrence of bfpA between those with and those without symptoms, yet, despite the high number of EPEC cases, there was only one TD with EPEC as the sole pathogen. It should be pointed out that some atypical EPEC strains harbour the bfpA gene, even though they do not express the pili [29]. It was not possible to systematically analyse the expression of a functional pilin in the current material afterwards. The expression is sensitive to *in vitro* conditions, and a carefully controlled study setup is required to confirm its presence [29]. Until now, typical EPEC has been considered to affect only children up to two years of age [25,27]. The molecular characteristics of EPEC associated with diarrhoea in adult travellers should be explored separately.

An impressive finding was the multitude and variety of pathogens in the faecal samples: in 78% of the travellers’ specimens two or more bacterial pathogens were identified, four of them with up to four pathogens. These findings can evidently be ascribed both to a substantial exposure to contaminated foods and the effective diagnostic methods used. The results accord with some previous reports [7,8,28] as well as our recent research [1], where 20- 60% of travellers to varying destinations have proved to carry multiple pathogens. Indeed, we also isolated bacterial pathogens in the stools of the small group of asymptomatic travellers: 67% had two or more bacterial pathogens, one had even four. The low number of asymptomatic participants (merely six) does not allow any definite conclusions from these data; an abundance of bacterial pathogens in asymptomatic travellers has, however, recently been reported in two other studies [5,8]. It should be pointed out that the stools of our volunteers were also explored for parasites with a newly developed qPCR method which has not been published as yet. The analysis showed that none of the volunteers had *Giardia lamblia*, *Cryptosporidium parvum*, or *Entamoeba histolytica* in their post-travel specimens (Juha Kirveskari, personal communication).

The major limitation of the present investigation was its low number of travellers. Nonetheless it represents one of the few TD studies carried out among a large group of travellers visiting the same place and residing in similar conditions for exactly the same period of time. Despite the homogeneous circumstances, the variety of pathogens proved large, the findings varying considerably between individual travellers.

The results of this investigation further attest to the need to improve routine diagnostics of TD. While merely 26% of our travellers having the disease (assuming that all EIEC/Shigella had been Shigella spp.) would have obtained a diagnosis using methods prevalent in routine diagnostics in most countries, the technique we applied revealed a pathogen in 92% of those with ongoing (n = 37) or recently subsided (n = 2) symptoms. In numerous earlier investigations [3] detecting just one pathogen in most TD patients, diarrhoeagenic *E. coli* (e.g. EAEC or EPEC) were not all examined, and even half of the stool samples proved negative for bacterial pathogens [3]. With these results in hand, we face the dilemma of how to determine the actual role of the various pathogens. The clinical characteristics of diseases caused by the various pathogens should each be re-examined in larger studies using modern methods.

## Conclusions

In the group of travellers to Benin, West Africa, 67% of contracted TD. EPEC, EAEC and ETEC were the most common findings with 79% of the cases having a co-infection. As modern diagnostics reveals in most patients a multitude of pathogens, the role of each pathogen should be re-evaluated.

## Abbreviations

EAEC: Enteroaggregative *Escherichia coli*; EHEC: Enterohaemorrhagic *Escherichia coli*; EIEC: Enteroinvasive *Escherichia coli*; EPEC: Enteropathogenic *Escherichia coli*; ETEC: Enterotoxigenic *Escherichia coli*; qPCR: Quantitative PCR; TD: Traveller’s diarrhoea.

## Competing interests

JA and JK have filed a patent application related to the qPCR method described in the paper. TL, SHP, JR and AK declare that they have no competing interests.

## Authors’ contribution

Study concept and design: JR, JK, AK; acquisition of data TL, SHP, JA, JR, JK, AK; analysis and interpretation of data TL, SHP, JA, SM, JK, AK; drafting of the manuscript TL, JK, AK; statistical analysis TL; final approval of the version published TL, SHP, JA, JR, SM, JK, AK. All authors read and approved the final manuscript.

## Authors’ information

MD TL is a specialist in internal medicine specialising in infectious diseases at the Helsinki University Central Hospital; MSc SHP is a researcher at the University of Helsinki; PhD JA is a clinical microbiologist at Helsinki University Hospital Laboratory; MD JR is a specialist in travel medicine in the Travel Clinic of Aava Medical centre; MSc SM is a clinical microbiologist in training at Helsinki University Hospital Laboratory; MD PhD JK is an specialist in clinical microbiology at Helsinki University Hospital Laboratory; Associate Professor AK is a specialist in internal medicine and in infectious diseases at the Helsinki University Central Hospital, and the Head of the Travel Clinic of Aava Medical Centre.

## Pre-publication history

The pre-publication history for this paper can be accessed here:

http://www.biomedcentral.com/1471-2334/14/81/prepub
